# HER2-low non-metastatic breast cancer in Qatar—a nationwide retrospective cohort study to evaluate the response to neoadjuvant chemotherapy: a real-world analysis

**DOI:** 10.3389/fonc.2024.1398100

**Published:** 2024-10-16

**Authors:** Ahmed Kardousha, Wafaa Shehada, Ahmed Basha, Sahar Nasser, Mufid El Mistiri, Anas Hamad, Salha Bujassoum Al-Bader, Shereen Elazzazy

**Affiliations:** ^1^ Pharmacy Department, National Center for Cancer Care and Research (NCCCR), Hamad Medical Corporation, Doha, Qatar; ^2^ Nursing Department, National Center for Cancer Care and Research (NCCCR), Hamad Medical Corporation, Doha, Qatar; ^3^ Medical Oncology Department, National Center for Cancer Care and Research (NCCCR), Hamad Medical Corporation, Doha, Qatar; ^4^ College of Pharmacy, QU Health, Qatar University (QU), Doha, Qatar

**Keywords:** breast cancer, Her2- low, neo-adjuvant chemotherapy, non-metastatic, real-world data

## Abstract

Globally, breast cancer is the most prevalent cancer and one of the leading causes of cancer-related death among women. HER2-low breast cancer represents a recently identified molecular category within breast cancer characterized by tumors displaying only slight overexpression of HER2 or lacking gene amplification. To illustrate, HER2-low tumors typically have an IHC (immune histochemistry) score of 1+ or 2+ with negative amplification. Nonetheless, recent findings indicate that even a slight amplification of HER2 could notably influence both therapeutic responses and prognostic outcomes. Our study aims to unveil the impact of HER2-low expression on the response to anthracycline and taxane-based neoadjuvant chemotherapy (NACT) in comparison to the HER2-negative group in non-metastatic breast cancer. This is a retrospective cohort study. All patients’ profiles with non-metastatic, HER2-low, and HER2-negative breast cancers who were administered neo-adjuvant chemotherapy and had surgery performed within the period spanning from 1 January 2018 to 30 August 2022 were enrolled. HER2-positive breast cancer patients were excluded. The evaluation of patients’ responses was conducted through the examination of surgical pathology reports to compare the two study groups (HER2-low and HER2-negative). The primary objective was evaluating the response to NACT comparing the objective response rate (ORR) in each of the two groups of HER2-low and HER2-negative patients. The total number of patients included was 262 patients; the majority were HER2-low 89% (233/262) vs. 11% (29/262) HER2-negative. An ORR (complete and partial response) to NACT was shown in 71% (185/262) of all patients. The ORR was similar in both groups, 70% (164/233) in the HER2-low group vs. 73% (21/29) in the HER2-negative group, with a statistical difference, OR: 1 (95% CI: 0.8–1), p-value 0.8. Similarly, the pathological complete response (pCR) rate was the same in both study groups at 14%, OR: 0.7 (95% CI: 0.2–3), p-value: 0.6. Interestingly, patients with hormone-positive tumors across both study groups had a higher response rate compared to hormone-negative patients. In the HER2-low cohort, the ORR was higher in patients with hormone-positive tumors in comparison with those with hormone-negative tumors [73% vs. 27%, OR: 0.8 (95% CI:0.8–1), p-value: 0.001]. Comparatively, in the HER2-negative cohort, ORR was also higher in patients with hormone-positive tumors compared to hormone-negative tumors [52% vs. 48%, OR: 2 (95% CI: 1–5), p-value: 0.05]. Subsequently, the ORR of all hormone-positive tumors with a positive outcome (CR or PR) was assessed categorizing the patients based on their HER2 expression. Concerning patients who expressed partial response (N = 115), a statistically significant difference was observed in HER2- low hormone-positive tumors as opposed to HER2-negative hormone-positive tumors [90% vs. 10%, OR: 0.7 (95% CI: 0.5–0.9), p-value: 0.001]. Remarkably, all patients with complete responses were from the HER2-low cohort. Our findings demonstrated a significant influence of HER2-low expression on the response to neoadjuvant chemotherapy among patients with hormone-positive HER2-low breast cancer within the studied cohort. Further studies are needed to evaluate the influence of hormonal expression on the response rate to NACT in the HER2-low patients in our population.

## Introduction

Globally, breast cancer (BC) has grown to become the most prevalent type of cancer according to recent statistics by the World Health Organization. BC currently accounts for approximately 12% of all newly diagnosed cancer cases globally with 2 million cases diagnosed in 2020. In the United States (U.S.), it is approximated that 30% of all new cases per annum are BC (1). Among those, 280,000 will be diagnosed at the advanced stage, while over 49,000 cases will be diagnosed in the early stages. Unfortunately, the lifetime risk of invasive BC is predicted at 13% for women in the U.S (1). Despite mortality rates steadily dropping over the years owing to many breakthroughs in BC screening and treatment, over 40,000 women in the U.S. have lost their lives to BC in 2022 (2).

Breast cancer is delineated into three distinct molecular subtypes as follows: hormone receptor-positive (HR+), human epidermal growth factor receptor 2 positive (HER2+), and triple-negative breast cancer (TNBC), the latter of which denotes the most clinically aggressive phenotype within the spectrum of breast malignancies (3). Typically, human epidermal growth factor receptor 2 (HER2) proteins regulate the growth, proliferation, and maintenance of breast cells (4, 5). In certain instances of breast cancer, breast cells exhibit elevated levels of HER2 on their cell surfaces leading to uncontrolled proliferation and division. Breast cancer patients are regularly screened for HER2 expression using a biochemical binding technique that allows visualization of HER2 protein. Tumors then get assigned an immunohistochemical (IHC) score. Currently, HER2 status classification bifurcates into either HER2-positive or -negative. HER2-positive breast cancers, which include tumors with an immunohistochemical score of 2+ with gene amplification or 3+, can be treated with HER2-targeted therapies such as trastuzumab.

Anti-HER2-targeted therapies have shifted the treatment paradigm of breast cancer significantly improving the prognosis for HER2-positive breast cancer patients after its approval in 1998 (6). The recognition of human epidermal growth factor receptor 2 (HER2) represented a significant breakthrough in the therapeutic approach to breast cancer heralding a transformative era in the management of HER2-positive tumors. Roughly 15% to 20% of breast cancer patients are characterized as HER2-positive, and their cancers have a poor prognosis, higher relapse rates, and higher rates of metastasis (7). Contrarywise, breast cancers exhibiting HER2 levels below the defined immunohistochemical threshold are ordinarily categorized as HER2-negative with no distinction of HER2-low subtype. Consequently, anti-HER2 therapies are not recommended (8).

This advancing concept garnered notable attention within the scientific community, particularly in response to the findings of recent clinical trials employing novel anti-HER2 targeted agents, such as trastuzumab–deruxtecan (9). Interestingly, these drugs showed antitumor efficacy in patients with HER2-low breast cancer as well as in those with HER2-positive breast cancer in a phenomenon known as the bystander-killing effect. Upon release, the potent cytotoxic deruxtecan payload permeates the cell membrane, inducing DNA damage and subsequent cell death, leading to the eradication of targeted tumor cells along with adjacent cells (9). In addition, this observation suggests the possibility that HER2-low and HER2-IHC 0 breast cancers could constitute distinct disease entities. However, existing data regarding the clinical disparities between these two groups are lacking, and a thorough understanding of the biology underlying HER2-low breast cancer remains to be attained. The term “HER2-low” was recently introduced following the DESTINY-Breast04 trial at the American Society of Clinical Oncology (ASCO) conference in 2022. Between 50% and 60% of all breast cancer patients have tumors with low HER2 expression. HER2-low breast cancer is a diverse population that includes both hormone receptor (HR) positive and hormone receptor (HR) negative breast cancers (9). Following the substantial findings of the DESTINY-Breast04 trial, more research efforts focusing on HER2-low breast cancers have been put forward. This led to the presentation of an even newer class of HER2-low tumors labeled as HER2-ultralow breast cancers. The idea of HER2-ultralow breast cancers (defined as IHC 0 with membrane staining in ≤10% of tumor cells) was lately conceptualized by the DESTINY-Breast06 trial presented at the 2024 annual ASCO conference (10).

As per the existing breast cancer guidelines, individuals diagnosed with HER2-low breast cancers continue to be classified as having HER2-negative breast cancer, for whom existing HER2-targeted therapies have not demonstrated efficacy and, thus, are not advised. New literature indicates a difference in chemotherapy response and overall survival in HER2-low breast cancers when compared to HER2-negative breast cancers (11–15). Several reported findings indicated that HER2-low tumors exhibited notably reduced rates of pathological complete response compared to HER2-zero tumors yet demonstrated significantly prolonged survival (12). Furthermore, an ongoing phase II clinical trial assessing the use of trastuzumab–deruxtecan in the neoadjuvant setting in HER2-low HR+ tumors reported that patients diagnosed with localized, hormone receptor-positive, HER2-low breast cancer and subjected to trastuzumab–deruxtecan treatment in the neoadjuvant setting demonstrated an overall response rate of 75% in the absence of anastrozole, and 63% when administered in conjunction with anastrozole. Such results shed light on the distinction between HER2-low and -negative breast cancers, the difference in response to chemotherapy between the two groups, and the possibility of anti-HER2-targeted therapy for HER2-low breast cancers.

Our study analyzed the impact of HER2-low expression on the response to NACT. The primary objective was to evaluate the response to NACT by comparing the ORR—defined as pathological complete response and pathological partial response—in each of the two HER2-low and -negative patient groups.

## Materials and methods

**Figure 1:**
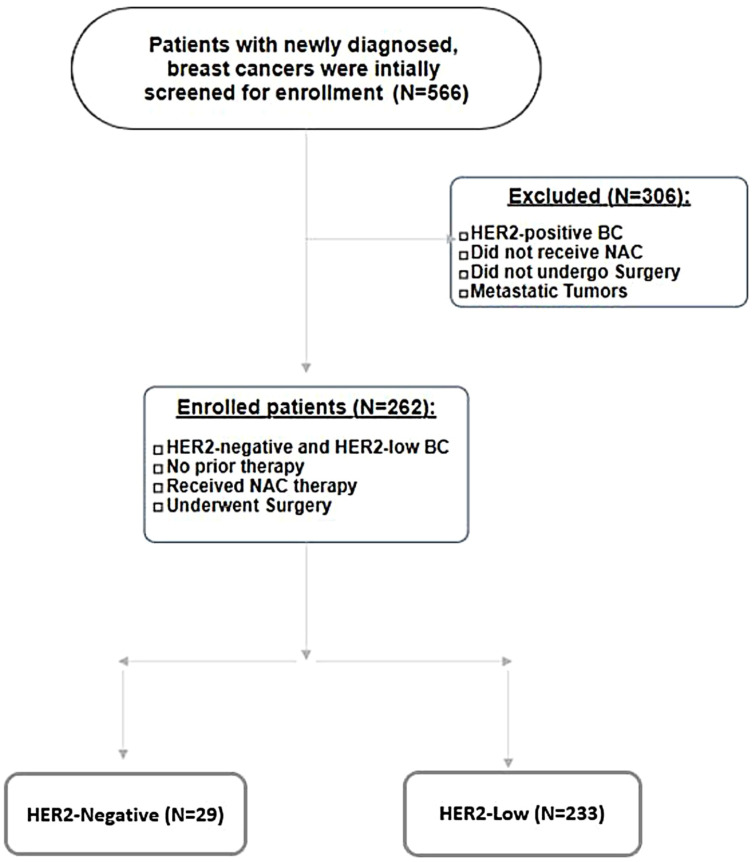
Patient selection flowchart.

We conducted a retrospective cohort study over a period of 56 months, from 1January 2018 until 30 August 2022. A total of 566 breast cancer patients were initially screened for inclusion. Inclusion criteria were newly diagnosed breast cancer cases, curative intent of treatment, HER2-low and -negative breast cancer subtypes including hormone-positive and -negative subtypes, systemic neoadjuvant chemotherapy, no prior therapy, and patients who underwent surgery. Exclusion criteria were HER2-positive tumors (IHC 3+ or IHC 2+ with amplification by FISH), patients who did not receive neoadjuvant chemotherapy and/or did not undergo surgery, and metastatic breast cancers. Based on the abovementioned criteria, 304 were excluded from the analysis, and 262 patients with non-metastatic, HER2-low, and HER2-negative breast cancers who were administered neo-adjuvant chemotherapy and had surgery performed within the period spanning from 1 January 2018 to 30 August 30 2022 were enrolled. Please refer to **Figure 1** (Patient Selection Flowchart).

Cancer staging was done in accordance with the eighth edition of the American Joint Committee on Cancer (AJCC) staging manual. Patients’ responses to neoadjuvant chemotherapy were clinically evaluated using surgical pathology reports to compare the two study groups. The primary objective was to collate the response to neoadjuvant chemotherapy reported as the ORR (pathologic complete and partial response) of HER2-low and -negative tumors. Pathologic complete response (pCR) is defined as the absence of invasive cancer and *in situ* cancer in the breast and axillary nodes, while pathologic partial response is described as at least a 30% reduction in the sum of diameters of target lesion in reference to baseline diameter sum. The secondary objective was to evaluate the impact of HR positivity on the response. A sub-group analysis compared the influence of hormonal expression (HR+/HR−) on the response in both study groups.

Relevant data were collected on an Excel sheet. The data were subjected to descriptive statistical analysis, wherein continuous variables were represented by means and standard deviations, while scale or nominal data were expressed as frequencies and percentages. Results were conveyed through tables and figures displaying frequencies and percentages. Patient characteristics were outlined using proportions, means, and standard deviations where applicable. The Chi-square test was employed to compare the frequencies of categorical variables between groups, while a two-tailed Student test was used for continuous variables comparison. Significant predictors were reported through odds ratio (OR), 95% confidence interval (CI), and p-value, with significance set at p < 0.05. All statistical analyses were conducted using SPSS 24 (SPSS Inc., Chicago, IL).

## Results

**Table 1** summarizes patient demographics, baseline clinical and pathological characteristics, and the treatments offered.

**Table 1:** Demographics, and baseline clinical and pathological characteristics.

Variable	N (%)
Age	
- 20–40	96 (37%)
- 40–60	144 (55%)
- Above 60	22 (8%)
Gender	
- Female	259 (99%)
- Male	3 (1%)
Stage	
- Early stage	198 (75%)
- Locally advanced	64 (25%)
Hormone status	
- Hormone positive	196 (75%)
- Hormone negative	66 (25%)
HER2, IHC	
- 0	29 (11%)
- 1+	95 (36%)
- 2+	138 (53%)
HER2-low	233(89%)
Neoadjuvant regimen*	
- FEC + D	109 (42%)
- AC+T	87 (33%)
- Carboplatin +paclitaxel + AC	35 (13%)
- Others	16 (6%)
- Dose-dense AC + T	6 (2.5%)
- TC	6 (2.5%)
- AC+D	3 (1%)

*AC, doxorubicin and cyclophosphamide; D, docetaxel; T, paclitaxel; FEC, fluorouracil, epirubicin, cyclophosphamide; TC, docetaxel and cyclophosphamide.

**Figure 2:**
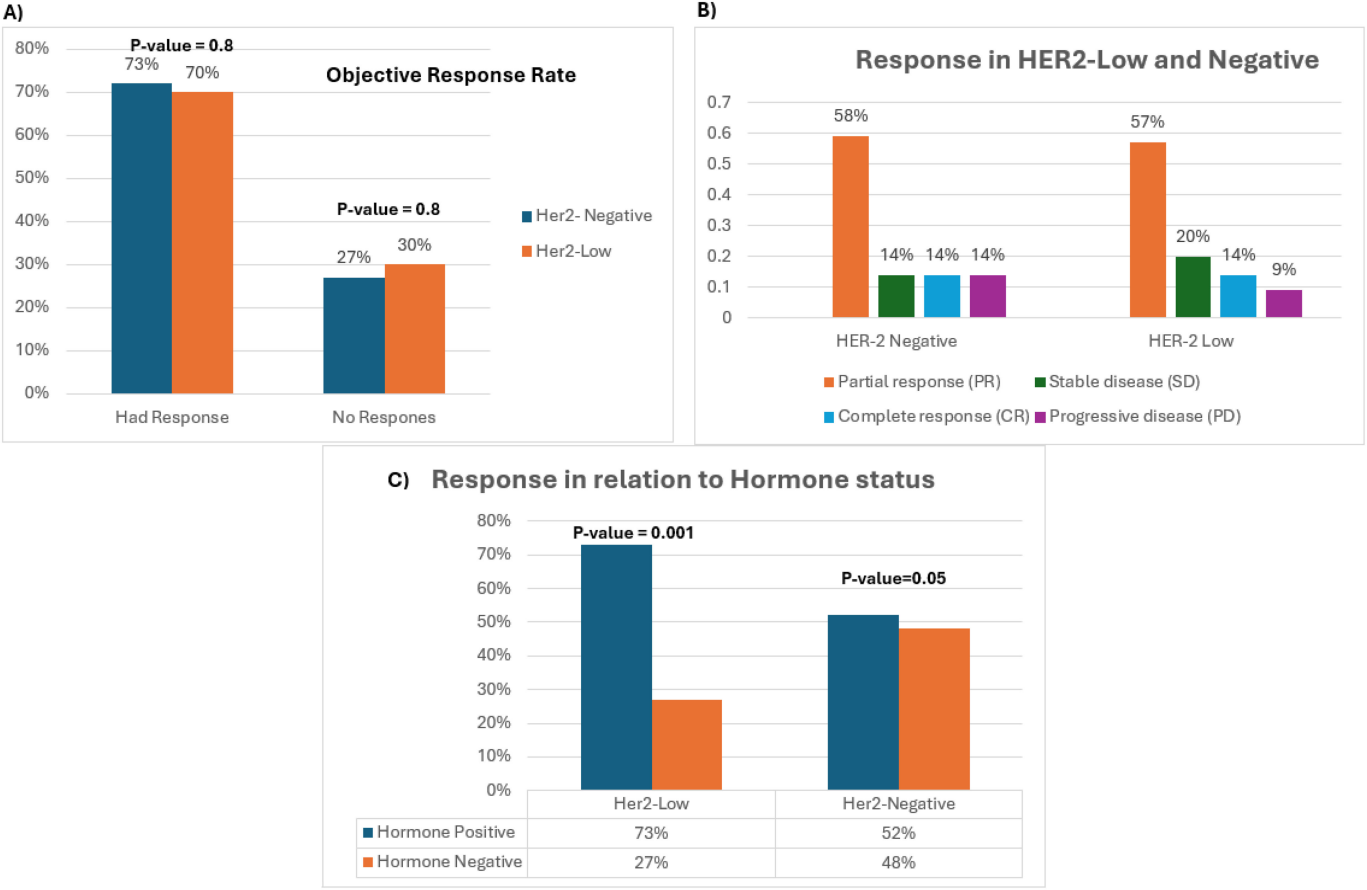
**(A)** Bar chart depicting total ORR and negative response of HER2-low and -negative tumors. **(B)** Bar chart representing the different types of response across both study groups. **(C)** Response of hormone-positive tumors compared to hormone-negative tumors across both study groups.

A total of 262 patients were included; the majority were HER2-low 89% (233/262) vs. 11% (29/262) HER2-negative. An ORR (complete and partial response) to NACT was shown in 71% (185/262) of all patients. The ORR rate was similar in both groups, 70% (164/233) in the HER2-low group vs. 73% (21/29) in the HER2-negative group, OR: 1 (95% CI: 0.8–1), p-value: 0.8 (**Figure 2A**). Similarly, the pathological complete response (pCR) rate was the same in both study groups at 14%, OR: 0.7 (95% CI: 0.2–3), p-value: 0.6 (**Figure 2B**). Hence, the unfavorable outcomes including stable disease and progression after NACT were also comparable in both groups at 27% (8/29) and 30% (69/233) in the HER2-negative and HER2-low groups, respectively, OR: 0.9 (95% CI: 0.5–1), p-value: 0.8 (**Figure 2A**).

With a focus on hormonal expression, patients with hormone-positive tumors across both study groups had a higher response rate compared to hormone-negative patients. However, statistical significance was shown in the HER2-low group only. In the HER2-low cohort, 73% of the patients (119/164) with hormone-positive tumors showed an ORR vs. 27% of patients (45/164) with hormone-negative tumors, OR: 0.8 (95% CI: 0.8–1), p-value: 0.001. Comparatively, in the HER2-negative cohort, 52% of the patients (11/21) with hormone-positive tumors had an ORR vs. 48% (10/21) with hormone-negative tumors, OR: 2 (95% CI: 1–5), p-value: 0.05 (**Figure 2C**). To accentuate our observation, the ORR of all patients with hormone-positive tumors who had a positive outcome was evaluated based on their HER2 expression (HER2-low vs. -negative). While all patients with complete response had HER2-low tumors(N = 15), 115 patients from both study groups had a partial response. Intriguingly, a statistically significant difference was detected in patients with HER2-low hormone-positive tumors relative to patients with HER2-negative hormone-positive tumors [90% vs. 10%, OR: 0.7 (95% CI: 0.5–0.9), p-value: 0.001] substantiating our claims.

## Discussion

Breast cancer is the most common cancer among women worldwide (2). The HER2 gene plays an important role in the development of breast cancer, as it is found to be amplified in approximately 15%–20% of breast cancer patients (7). HER2 is a transmembrane receptor tyrosine kinase that regulates the growth and proliferation of breast cells. The amplification and overexpression of HER2 in HER2-positive breast cancer are correlated with heightened disease aggressiveness and inferior prognostic outcomes compared to HER2-negative breast cancer (16). The extent and the complexity of cancer’s diversity, which includes genetics, tumor heterogeneity, and resistance to therapy, are challenging (17). Despite the remarkable progress in alternative therapies for breast cancer, chemotherapy remains one of the main treatment pillars, especially for patients with late-stage diseases. The survival rate of BC patients in some developed countries reaches up to 80% due to early detection and treatment (18). Great improvements have been achieved in understanding BC and developing prevention strategies in the last decade. BC is well classified, and several chemotherapeutic agents, targeted therapies, and immune therapies have been approved and shown to increase the overall survival and quality of life of BC patients.

However, recent research has shed light on a new subtype of breast cancer: HER2-low breast cancer. This subtype is defined as having low expression levels of the HER2 protein and has been found to make up approximately 60%–70% of all breast cancer cases (8). HER2-low breast cancer is believed to be distinct from HER2-positive breast cancer in terms of its molecular profile, clinical characteristics, and response to treatment.

The treatment of HER2-low breast cancer is an area of active research, and there is currently no consensus on the optimal treatment approach in non-metastatic disease. Treatment of this subgroup has been the same as that for HER2-negative breast cancer and includes the use of endocrine therapies for hormone-positive tumors, and chemotherapy. While anti HER2-targeted therapies are yet to be investigated. Recent studies have also shown a different response to chemotherapy where HER2-low breast cancer patients who received adjuvant chemotherapy have better outcomes than those who did not (19). Several studies have investigated the clinical characteristics of HER2-low breast cancer and compared them to other subtypes of breast cancer.

A national study from the Korean Breast Cancer Society analyzed the clinical significance of HER2-low expression in early breast cancer and discovered that HER2-low breast cancer exhibited a notable association with a decreased incidence of G3 tumors, diminished Ki-67 labeling index, and fewer TP53 mutations in comparison to HER2-negative (IHC 0) breast cancer (15). Similarly, another study from a single institution in China analyzed the clinicopathological characteristics and prognosis of HER2-low early-stage breast cancer and reported that HER2-low expression was related to better overall survival (OS) (20). These findings indicate that HER2-low breast cancer might exhibit a comparatively less aggressive phenotype when contrasted with other subtypes of breast cancer.

Moreover, recent studies have suggested that HER2-low breast cancer may have different responses to treatment compared to other subtypes of breast cancer. A study conducted in China juxtaposed the clinical and pathological attributes alongside the response to neoadjuvant systemic therapy between patients diagnosed with HER2-low and -negative breast cancer. The research revealed that HER2-low tumors exhibited a reduced ratio of pathological complete response (pCR) in comparison to HER2-negative tumors hinting at a potential association between HER2-low status and resistance to chemotherapy (21). Furthermore, according to a study conducted in Japan, HER2-low breast cancer was observed to have a poorer prognosis in HR-positive patients compared to HER2-negative breast cancer, whereas no significant difference was noted in HR-negative patients (22). This study aimed to assess the prevalence of diminished HER2 expression in breast cancer and to compare the prognostic outcomes among patients with HER2-low and HER2-negative breast cancer categorized based on hormone receptor (HR) status.

Overall, these findings suggest that HER2-low breast cancer may have different responses to chemotherapy and endocrine therapy compared to other subtypes of breast cancer.

Furthermore, a combined examination of individual patient data from four prospective neoadjuvant clinical trials, encompassing a cohort of 2,310 patients diagnosed with HER2-non-amplified primary breast cancer and treated with neoadjuvant chemotherapy, revealed that tumors characterized by HER2-low-positive expression exhibited a notably reduced rate of pathological complete response compared to HER2-negative tumors (12). The study also reported that pathological complete response was significantly lower in HER2-low-positive tumors versus HER2-zero tumors in the hormone receptor-positive subgroup but not in the hormone receptor-negative subgroup. Patients with HER2-low tumors also displayed significantly longer survival than did patients with HER2-negative tumors.

Recently, the FDA authorized the use of fam-trastuzumab–deruxtecan for the treatment of HER2-low unresectable or metastatic breast cancer following the outcomes of the DESTINY-Breast04 trial. The trial showed that fam-trastuzumab–deruxtecan had a higher objective response rate and a longer progression-free survival compared to chemotherapy in patients with HER2-low breast cancer. Likewise, a statistical and clinical significance was observed in the progression-free survival of all patient populations including HER2-low and -ultralow in the metastatic breast cancer setting as recently demonstrated by the preliminary results of DESTINY-Breast06 phase III trial (10). This implies that emerging anti-HER2-targeted treatments could be efficacious for managing HER2-low breast cancer; however, additional investigation is warranted to ascertain the most suitable treatment strategy.

Taking the published literature into consideration, our retrospective analysis evaluated the impact of HER2-low expression on the response to neoadjuvant chemotherapy when compared to HER2-negative breast cancer. Although more patients in the HER2-low hormone-positive population had a response to NACT compared to the HER2-low hormone-negative population, there was no statistically significant difference between HER2-low and negative tumors among the overall population. Our results support the conclusion that the HER2-low HR-positive cohort significantly benefited from the traditional chemotherapy in the neoadjuvant settings. Nevertheless, the HER2-low HR-negative cohort is unlikely to benefit from traditional chemotherapy compared to the HER2-negative HR-negative cohort. Still, newer anti-HER2 targeted therapies, like trastuzumab–deruxtecan might have a role in this particular patient population, and further research is required to validate and extrapolate the results of the DESTINY-Breast04 and DESTINY-Breast06 trials.

Our study had several limitations. First, the retrospective design had associated potential biases. Second, the sample size was relatively small; however, we included all patients diagnosed during the study period. In addition, there was an uneven distribution of patients in both study groups. The high number of patients in the HER2-low group compared to the HER2-negative group created an imbalance between the two study groups that might have impacted the results.

Conclusively, HER2-low is a newly defined molecular subtype of breast cancer with low levels of HER2 expression that may have distinct clinical and biological characteristics compared to other subtypes of breast cancer. Recent studies have suggested that HER2-low breast cancer may have different responses to treatment compared to other subtypes of breast cancer, and there is ongoing research to identify the optimal treatment strategy for this subgroup. The biology of HER2-low breast cancer is still poorly understood, and further research is needed to identify the molecular mechanisms underlying this subtype. The encouraging effectiveness of innovative HER2-targeted therapy in treating advanced HER2-low breast cancers has prompted consideration for revising the clinical classification of HER2 status in breast cancer to incorporate a HER2-low category. However, its efficacy in early-stage breast cancer remains under investigation.

## Conclusion

Our study discerned no statistically significant variance in neoadjuvant chemotherapy response between patients with HER2-low and HER2-negative breast cancer. However, we observed a significant association between HER2-low expression, hormonal status, and neoadjuvant chemotherapy response in non-metastatic breast cancer.

## Data Availability

The raw data supporting the conclusions of this article will be made available by the authors, without undue reservation.
